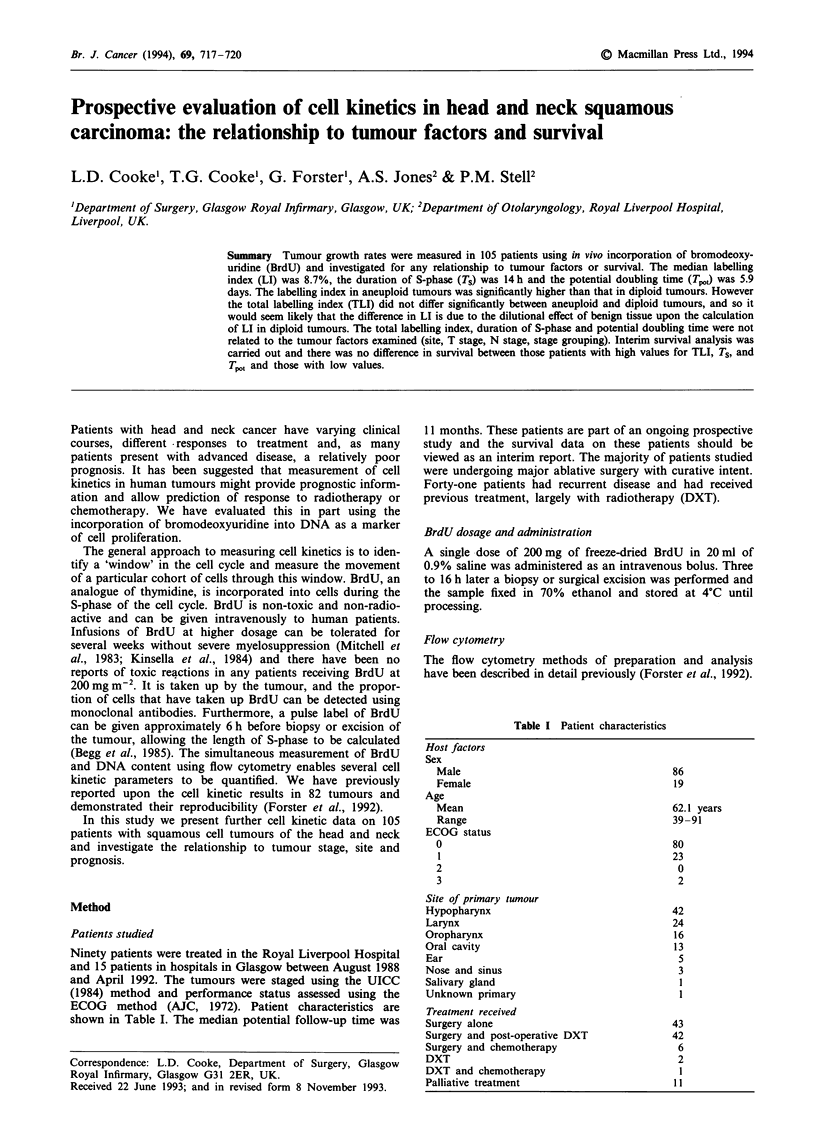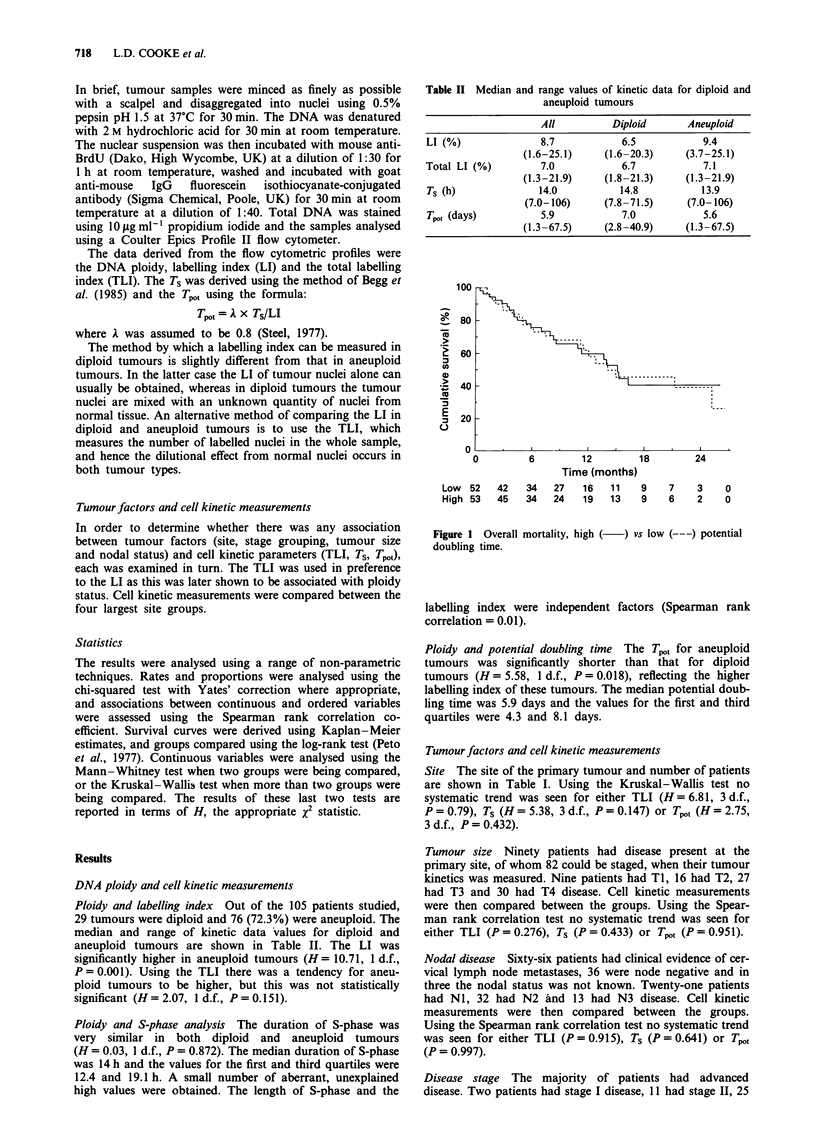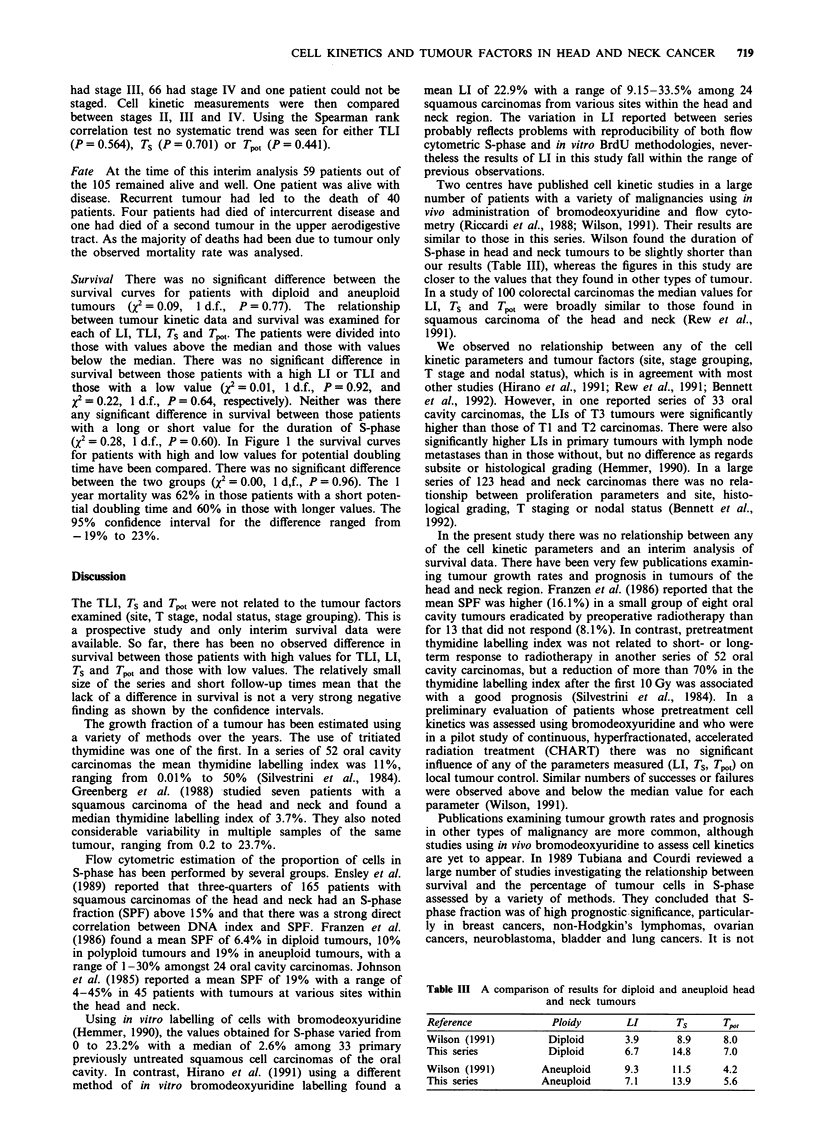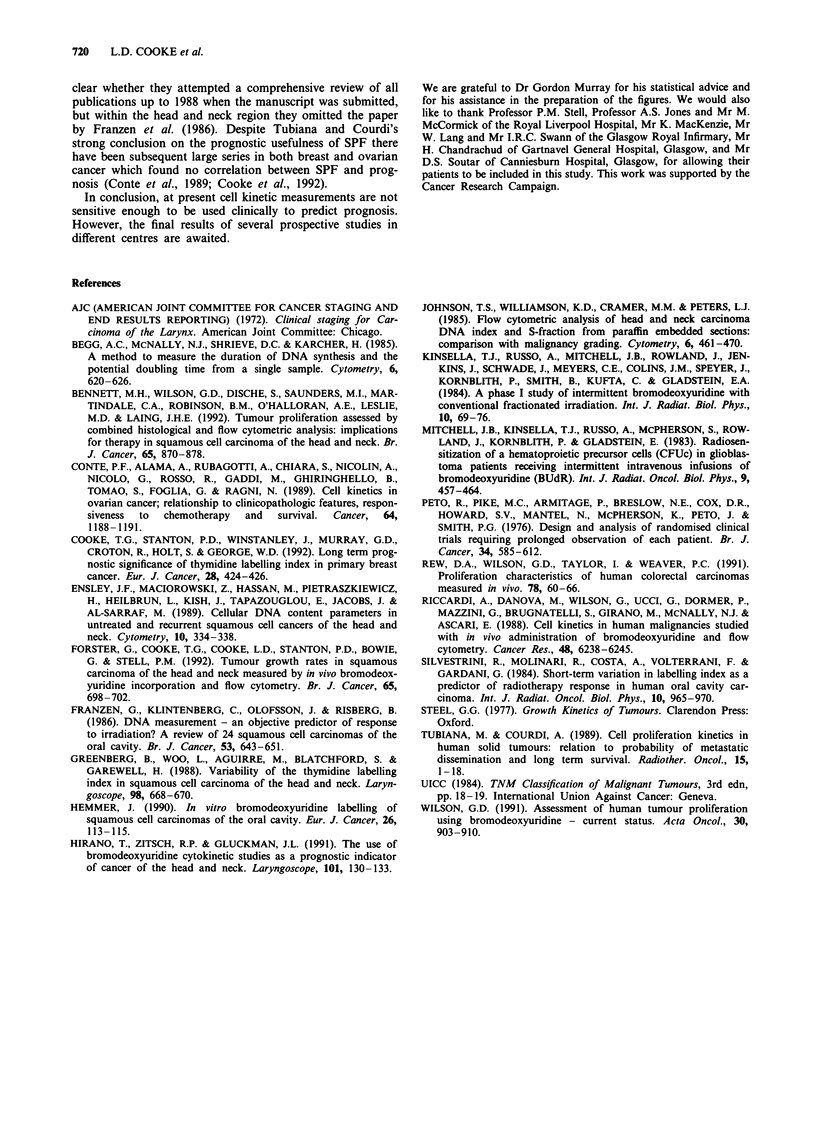# Prospective evaluation of cell kinetics in head and neck squamous carcinoma: the relationship to tumour factors and survival.

**DOI:** 10.1038/bjc.1994.135

**Published:** 1994-04

**Authors:** L. D. Cooke, T. G. Cooke, G. Forster, A. S. Jones, P. M. Stell

**Affiliations:** Department of Surgery, Glasgow Royal Infirmary, UK.

## Abstract

Tumour growth rates were measured in 105 patients using in vivo incorporation of bromodeoxyuridine (BrdU) and investigated for any relationship to tumour factors or survival. The median labelling index (LI) was 8.7%, the duration of S-phase (Ts) was 14 h and the potential doubling time (Tpot) was 5.9 days. The labelling index in aneuploid tumours was significantly higher than that in diploid tumours. However the total labelling index (TLI) did not differ significantly between aneuploid and diploid tumours, and so it would seem likely that the difference in LI is due to the dilutional effect of benign tissue upon the calculation of LI in diploid tumours. The total labelling index, duration of S-phase and potential doubling time were not related to the tumour factors examined (site, T stage, N stage, stage grouping). Interim survival analysis was carried out and there was no difference in survival between those patients with high values for TLI, Ts, and Tpot and those with low values.


Br. J. Cancer (1994), 69, 717-720 © Macmillan Press Ltd., 1994

### Prospective evaluation of cell kinetics in head and neck squamous carcinoma: the relationship to tumour factors and survival

L.D. Cooke[1], T.G. Cooke[1], G. Forster[1], A.S. Jones[2] & P.M. Stell[2]

[1]*Department of Surgery, Glasgow Royal Infirmary, Glasgow, UK;* [2]*Department of Otolaryngology, Royal Liverpool Hospital, Liverpool, UK.*

**Summary** Tumour growth rates were measured in 105 patients using *in vivo* incorporation of bromodeoxyuridine (BrdU) and investigated for any relationship to tumour factors or survival. The median labelling index (LI) was 8.7%, the duration of S-phase ($T_S$) was 14 h and the potential doubling time ($T_{pot}$) was 5.9 days. The labelling index in aneuploid tumours was significantly higher than that in diploid tumours. However the total labelling index (TLI) did not differ significantly between aneuploid and diploid tumours, and so it would seem likely that the difference in LI is due to the dilutional effect of benign tissue upon the calculation of LI in diploid tumours. The total labelling index, duration of S-phase and potential doubling time were not related to the tumour factors examined (site, T stage, N stage, stage grouping). Interim survival analysis was carried out and there was no difference in survival between those patients with high values for TLI, $T_S$, and $T_{pot}$ and those with low values.

Patients with head and neck cancer have varying clinical courses, different responses to treatment and, as many patients present with advanced disease, a relatively poor prognosis. It has been suggested that measurement of cell kinetics in human tumours might provide prognostic information and allow prediction of response to radiotherapy or chemotherapy. We have evaluated this in part using the incorporation of bromodeoxyuridine into DNA as a marker of cell proliferation.

The general approach to measuring cell kinetics is to identify a 'window' in the cell cycle and measure the movement of a particular cohort of cells through this window. BrdU, an analogue of thymidine, is incorporated into cells during the S-phase of the cell cycle. BrdU is non-toxic and non-radioactive and can be given intravenously to human patients. Infusions of BrdU at higher dosage can be tolerated for several weeks without severe myelosuppression (Mitchell *et al.*, 1983; Kinsella *et al.*, 1984) and there have been no reports of toxic reactions in any patients receiving BrdU at 200 mg $m^{-2}$. It is taken up by the tumour, and the proportion of cells that have taken up BrdU can be detected using monoclonal antibodies. Furthermore, a pulse label of BrdU can be given approximately 6 h before biopsy or excision of the tumour, allowing the length of S-phase to be calculated (Begg *et al.*, 1985). The simultaneous measurement of BrdU and DNA content using flow cytometry enables several cell kinetic parameters to be quantified. We have previously reported upon the cell kinetic results in 82 tumours and demonstrated their reproducibility (Forster *et al.*, 1992).

In this study we present further cell kinetic data on 105 patients with squamous cell tumours of the head and neck and investigate the relationship to tumour stage, site and prognosis.

### Method

### *Patients studied*

Ninety patients were treated in the Royal Liverpool Hospital and 15 patients in hospitals in Glasgow between August 1988 and April 1992. The tumours were staged using the UICC (1984) method and performance status assessed using the ECOG method (AJC, 1972). Patient characteristics are shown in Table I. The median potential follow-up time was 11 months. These patients are part of an ongoing prospective study and the survival data on these patients should be viewed as an interim report. The majority of patients studied were undergoing major ablative surgery with curative intent. Forty-one patients had recurrent disease and had received previous treatment, largely with radiotherapy (DXT).

### *BrdU dosage and administration*

A single dose of 200 mg of freeze-dried BrdU in 20 ml of 0.9% saline was administered as an intravenous bolus. Three to 16 h later a biopsy or surgical excision was performed and the sample fixed in 70% ethanol and stored at 4°C until processing.

### *Flow cytometry*

The flow cytometry methods of preparation and analysis have been described in detail previously (Forster *et al.*, 1992).

**Table I** Patient characteristics

| | |
|---|---|
| *Host factors* | |
| Sex | |
| Male | 86 |
| Female | 19 |
| Age | |
| Mean | 62.1 years |
| Range | 39-91 |
| ECOG status | |
| 0 | 80 |
| 1 | 23 |
| 2 | 0 |
| 3 | 2 |
| *Site of primary tumour* | |
| Hypopharynx | 42 |
| Larynx | 24 |
| Oropharynx | 16 |
| Oral cavity | 13 |
| Ear | 5 |
| Nose and sinus | 3 |
| Salivary gland | 1 |
| Unknown primary | 1 |
| *Treatment received* | |
| Surgery alone | 43 |
| Surgery and post-operative DXT | 42 |
| Surgery and chemotherapy | 6 |
| DXT | 2 |
| DXT and chemotherapy | 1 |
| Palliative treatment | 11 |

Correspondence: L.D. Cooke, Department of Surgery, Glasgow Royal Infirmary, Glasgow G31 2ER, UK.
Received 22 June 1993; and in revised form 8 November 1993.

In brief, tumour samples were minced as finely as possible with a scalpel and disaggregated into nuclei using 0.5% pepsin pH 1.5 at 37°C for 30 min. The DNA was denatured with 2 M hydrochloric acid for 30 min at room temperature. The nuclear suspension was then incubated with mouse anti-BrdU (Dako, High Wycombe, UK) at a dilution of 1:30 for 1 h at room temperature, washed and incubated with goat anti-mouse IgG fluorescein isothiocyanate-conjugated antibody (Sigma Chemical, Poole, UK) for 30 min at room temperature at a dilution of 1:40. Total DNA was stained using 10 $\mu$g ml$^{-1}$ propidium iodide and the samples analysed using a Coulter Epics Profile II flow cytometer.

The data derived from the flow cytometric profiles were the DNA ploidy, labelling index (LI) and the total labelling index (TLI). The $T_S$ was derived using the method of Begg *et al.* (1985) and the $T_{pot}$ using the formula:

$$T_{pot} = \lambda \times T_S/\text{LI}$$

where $\lambda$ was assumed to be 0.8 (Steel, 1977).

The method by which a labelling index can be measured in diploid tumours is slightly different from that in aneuploid tumours. In the latter case the LI of tumour nuclei alone can usually be obtained, whereas in diploid tumours the tumour nuclei are mixed with an unknown quantity of nuclei from normal tissue. An alternative method of comparing the LI in diploid and aneuploid tumours is to use the TLI, which measures the number of labelled nuclei in the whole sample, and hence the dilutional effect from normal nuclei occurs in both tumour types.

*Tumour factors and cell kinetic measurements*

In order to determine whether there was any association between tumour factors (site, stage grouping, tumour size and nodal status) and cell kinetic parameters (TLI, $T_S$, $T_{pot}$), each was examined in turn. The TLI was used in preference to the LI as this was later shown to be associated with ploidy status. Cell kinetic measurements were compared between the four largest site groups.

*Statistics*

The results were analysed using a range of non-parametric techniques. Rates and proportions were analysed using the chi-squared test with Yates' correction where appropriate, and associations between continuous and ordered variables were assessed using the Spearman rank correlation coefficient. Survival curves were derived using Kaplan-Meier estimates, and groups compared using the log-rank test (Peto *et al.*, 1977). Continuous variables were analysed using the Mann-Whitney test when two groups were being compared, or the Kruskal-Wallis test when more than two groups were being compared. The results of these last two tests are reported in terms of $H$, the appropriate $\chi^2$ statistic.

### Results

*DNA ploidy and cell kinetic measurements*

*Ploidy and labelling index* Out of the 105 patients studied, 29 tumours were diploid and 76 (72.3%) were aneuploid. The median and range of kinetic data values for diploid and aneuploid tumours are shown in Table II. The LI was significantly higher in aneuploid tumours ($H$ = 10.71, 1 d.f., $P$ = 0.001). Using the TLI there was a tendency for aneuploid tumours to be higher, but this was not statistically significant ($H$ = 2.07, 1 d.f., $P$ = 0.151).

*Ploidy and S-phase analysis* The duration of S-phase was very similar in both diploid and aneuploid tumours ($H$ = 0.03, 1 d.f., $P$ = 0.872). The median duration of S-phase was 14 h and the values for the first and third quartiles were 12.4 and 19.1 h. A small number of aberrant, unexplained high values were obtained. The length of S-phase and the labelling index were independent factors (Spearman rank correlation = 0.01).

**Table II** Median and range values of kinetic data for diploid and aneuploid tumours

| | *All* | *Diploid* | *Aneuploid* |
|---|---|---|---|
| LI (%) | 8.7<br>(1.6–25.1) | 6.5<br>(1.6–20.3) | 9.4<br>(3.7–25.1) |
| Total LI (%) | 7.0<br>(1.3–21.9) | 6.7<br>(1.8–21.3) | 7.1<br>(1.3–21.9) |
| $T_S$ (h) | 14.0<br>(7.0–106) | 14.8<br>(7.8–71.5) | 13.9<br>(7.0–106) |
| $T_{pot}$ (days) | 5.9<br>(1.3–67.5) | 7.0<br>(2.8–40.9) | 5.6<br>(1.3–67.5) |

**Figure 1** Overall mortality, high (——) *vs* low (---) potential doubling time.

*Ploidy and potential doubling time* The $T_{pot}$ for aneuploid tumours was significantly shorter than that for diploid tumours ($H$ = 5.58, 1 d.f., $P$ = 0.018), reflecting the higher labelling index of these tumours. The median potential doubling time was 5.9 days and the values for the first and third quartiles were 4.3 and 8.1 days.

*Tumour factors and cell kinetic measurements*

*Site* The site of the primary tumour and number of patients are shown in Table I. Using the Kruskal-Wallis test no systematic trend was seen for either TLI ($H$ = 6.81, 3 d.f., $P$ = 0.79), $T_S$ ($H$ = 5.38, 3 d.f., $P$ = 0.147) or $T_{pot}$ ($H$ = 2.75, 3 d.f., $P$ = 0.432).

*Tumour size* Ninety patients had disease present at the primary site, of whom 82 could be staged, when their tumour kinetics was measured. Nine patients had T1, 16 had T2, 27 had T3 and 30 had T4 disease. Cell kinetic measurements were then compared between the groups. Using the Spearman rank correlation test no systematic trend was seen for either TLI ($P$ = 0.276), $T_S$ ($P$ = 0.433) or $T_{pot}$ ($P$ = 0.951).

*Nodal disease* Sixty-six patients had clinical evidence of cervical lymph node metastases, 36 were node negative and in three the nodal status was not known. Twenty-one patients had N1, 32 had N2 and 13 had N3 disease. Cell kinetic measurements were then compared between the groups. Using the Spearman rank correlation test no systematic trend was seen for either TLI ($P$ = 0.915), $T_S$ ($P$ = 0.641) or $T_{pot}$ ($P$ = 0.997).

*Disease stage* The majority of patients had advanced disease. Two patients had stage I disease, 11 had stage II, 25

had stage III, 66 had stage IV and one patient could not be staged. Cell kinetic measurements were then compared between stages II, III and IV. Using the Spearman rank correlation test no systematic trend was seen for either TLI ($P = 0.564$), $T_S$ ($P = 0.701$) or $T_{pot}$ ($P = 0.441$).

*Fate* At the time of this interim analysis 59 patients out of the 105 remained alive and well. One patient was alive with disease. Recurrent tumour had led to the death of 40 patients. Four patients had died of intercurrent disease and one had died of a second tumour in the upper aerodigestive tract. As the majority of deaths had been due to tumour only the observed mortality rate was analysed.

*Survival* There was no significant difference between the survival curves for patients with diploid and aneuploid tumours ($\chi^2 = 0.09$, 1 d.f., $P = 0.77$). The relationship between tumour kinetic data and survival was examined for each of LI, TLI, $T_S$ and $T_{pot}$. The patients were divided into those with values above the median and those with values below the median. There was no significant difference in survival between those patients with a high LI or TLI and those with a low value ($\chi^2 = 0.01$, 1 d.f., $P = 0.92$, and $\chi^2 = 0.22$, 1 d.f., $P = 0.64$, respectively). Neither was there any significant difference in survival between those patients with a long or short value for the duration of S-phase ($\chi^2 = 0.28$, 1 d.f., $P = 0.60$). In Figure 1 the survival curves for patients with high and low values for potential doubling time have been compared. There was no significant difference between the two groups ($\chi^2 = 0.00$, 1 d,f., $P = 0.96$). The 1 year mortality was 62% in those patients with a short potential doubling time and 60% in those with longer values. The 95% confidence interval for the difference ranged from −19% to 23%.

### Discussion

The TLI, $T_S$ and $T_{pot}$ were not related to the tumour factors examined (site, T stage, nodal status, stage grouping). This is a prospective study and only interim survival data were available. So far, there has been no observed difference in survival between those patients with high values for TLI, LI, $T_S$ and $T_{pot}$ and those with low values. The relatively small size of the series and short follow-up times mean that the lack of a difference in survival is not a very strong negative finding as shown by the confidence intervals.

The growth fraction of a tumour has been estimated using a variety of methods over the years. The use of tritiated thymidine was one of the first. In a series of 52 oral cavity carcinomas the mean thymidine labelling index was 11%, ranging from 0.01% to 50% (Silvestrini *et al.*, 1984). Greenberg *et al.* (1988) studied seven patients with a squamous carcinoma of the head and neck and found a median thymidine labelling index of 3.7%. They also noted considerable variability in multiple samples of the same tumour, ranging from 0.2 to 23.7%.

Flow cytometric estimation of the proportion of cells in S-phase has been performed by several groups. Ensley *et al.* (1989) reported that three-quarters of 165 patients with squamous carcinomas of the head and neck had an S-phase fraction (SPF) above 15% and that there was a strong direct correlation between DNA index and SPF. Franzen *et al.* (1986) found a mean SPF of 6.4% in diploid tumours, 10% in polyploid tumours and 19% in aneuploid tumours, with a range of 1–30% amongst 24 oral cavity carcinomas. Johnson *et al.* (1985) reported a mean SPF of 19% with a range of 4–45% in 45 patients with tumours at various sites within the head and neck.

Using *in vitro* labelling of cells with bromodeoxyuridine (Hemmer, 1990), the values obtained for S-phase varied from 0 to 23.2% with a median of 2.6% among 33 primary previously untreated squamous cell carcinomas of the oral cavity. In contrast, Hirano *et al.* (1991) using a different method of *in vitro* bromodeoxyuridine labelling found a mean LI of 22.9% with a range of 9.15–33.5% among 24 squamous carcinomas from various sites within the head and neck region. The variation in LI reported between series probably reflects problems with reproducibility of both flow cytometric S-phase and *in vitro* BrdU methodologies, nevertheless the results of LI in this study fall within the range of previous observations.

Two centres have published cell kinetic studies in a large number of patients with a variety of malignancies using *in vivo* administration of bromodeoxyuridine and flow cytometry (Riccardi *et al.*, 1988; Wilson, 1991). Their results are similar to those in this series. Wilson found the duration of S-phase in head and neck tumours to be slightly shorter than our results (Table III), whereas the figures in this study are closer to the values that they found in other types of tumour. In a study of 100 colorectal carcinomas the median values for LI, $T_S$ and $T_{pot}$ were broadly similar to those found in squamous carcinoma of the head and neck (Rew *et al.*, 1991).

We observed no relationship between any of the cell kinetic parameters and tumour factors (site, stage grouping, T stage and nodal status), which is in agreement with most other studies (Hirano *et al.*, 1991; Rew *et al.*, 1991; Bennett *et al.*, 1992). However, in one reported series of 33 oral cavity carcinomas, the LIs of T3 tumours were significantly higher than those of T1 and T2 carcinomas. There were also significantly higher LIs in primary tumours with lymph node metastases than in those without, but no difference as regards subsite or histological grading (Hemmer, 1990). In a large series of 123 head and neck carcinomas there was no relationship between proliferation parameters and site, histological grading, T staging or nodal status (Bennett *et al.*, 1992).

In the present study there was no relationship between any of the cell kinetic parameters and an interim analysis of survival data. There have been very few publications examining tumour growth rates and prognosis in tumours of the head and neck region. Franzen *et al.* (1986) reported that the mean SPF was higher (16.1%) in a small group of eight oral cavity tumours eradicated by preoperative radiotherapy than for 13 that did not respond (8.1%). In contrast, pretreatment thymidine labelling index was not related to short- or long-term response to radiotherapy in another series of 52 oral cavity carcinomas, but a reduction of more than 70% in the thymidine labelling index after the first 10 Gy was associated with a good prognosis (Silvestrini *et al.*, 1984). In a preliminary evaluation of patients whose pretreatment cell kinetics was assessed using bromodeoxyuridine and who were in a pilot study of continuous, hyperfractionated, accelerated radiation treatment (CHART) there was no significant influence of any of the parameters measured (LI, $T_S$, $T_{pot}$) on local tumour control. Similar numbers of successes or failures were observed above and below the median value for each parameter (Wilson, 1991).

Publications examining tumour growth rates and prognosis in other types of malignancy are more common, although studies using *in vivo* bromodeoxyuridine to assess cell kinetics are yet to appear. In 1989 Tubiana and Courdi reviewed a large number of studies investigating the relationship between survival and the percentage of tumour cells in S-phase assessed by a variety of methods. They concluded that S-phase fraction was of high prognostic significance, particularly in breast cancers, non-Hodgkin's lymphomas, ovarian cancers, neuroblastoma, bladder and lung cancers. It is not

**Table III** A comparison of results for diploid and aneuploid head and neck tumours

| *Reference* | *Ploidy* | *LI* | $T_S$ | $T_{pot}$ |
|---|---|---|---|---|
| Wilson (1991) | Diploid | 3.9 | 8.9 | 8.0 |
| This series | Diploid | 6.7 | 14.8 | 7.0 |
| Wilson (1991) | Aneuploid | 9.3 | 11.5 | 4.2 |
| This series | Aneuploid | 7.1 | 13.9 | 5.6 |

clear whether they attempted a comprehensive review of all publications up to 1988 when the manuscript was submitted, but within the head and neck region they omitted the paper by Franzen *et al.* (1986). Despite Tubiana and Courdi's strong conclusion on the prognostic usefulness of SPF there have been subsequent large series in both breast and ovarian cancer which found no correlation between SPF and prognosis (Conte *et al.*, 1989; Cooke *et al.*, 1992).

In conclusion, at present cell kinetic measurements are not sensitive enough to be used clinically to predict prognosis. However, the final results of several prospective studies in different centres are awaited.

We are grateful to Dr Gordon Murray for his statistical advice and for his assistance in the preparation of the figures. We would also like to thank Professor P.M. Stell, Professor A.S. Jones and Mr M. McCormick of the Royal Liverpool Hospital, Mr K. MacKenzie, Mr W. Lang and Mr I.R.C. Swann of the Glasgow Royal Infirmary, Mr H. Chandrachud of Gartnavel General Hospital, Glasgow, and Mr D.S. Soutar of Canniesburn Hospital, Glasgow, for allowing their patients to be included in this study. This work was supported by the Cancer Research Campaign.

### References

AJC (AMERICAN JOINT COMMITTEE FOR CANCER STAGING AND END RESULTS REPORTING) (1972). *Clinical staging for Carcinoma of the Larynx*. American Joint Committee: Chicago.

BEGG, A.C., MCNALLY, N.J., SHRIEVE, D.C. & KARCHER, H. (1985). A method to measure the duration of DNA synthesis and the potential doubling time from a single sample. *Cytometry*, **6**, 620-626.

BENNETT, M.H., WILSON, G.D., DISCHE, S., SAUNDERS, M.I., MARTINDALE, C.A., ROBINSON, B.M., O'HALLORAN, A.E., LESLIE, M.D. & LAING, J.H.E. (1992). Tumour proliferation assessed by combined histological and flow cytometric analysis: implications for therapy in squamous cell carcinoma of the head and neck. *Br. J. Cancer*, **65**, 870-878.

CONTE, P.F., ALAMA, A., RUBAGOTTI, A., CHIARA, S., NICOLIN, A., NICOLO, G., ROSSO, R., GADDI, M., GHIRINGHELLO, B., TOMAO, S., FOGLIA, G. & RAGNI, N. (1989). Cell kinetics in ovarian cancer; relationship to clinicopathologic features, responsiveness to chemotherapy and survival. *Cancer*, **64**, 1188-1191.

COOKE, T.G., STANTON, P.D., WINSTANLEY, J., MURRAY, G.D., CROTON, R., HOLT, S. & GEORGE, W.D. (1992). Long term prognostic significance of thymidine labelling index in primary breast cancer. *Eur. J. Cancer*, **28**, 424-426.

ENSLEY, J.F., MACIOROWSKI, Z., HASSAN, M., PIETRASZKIEWICZ, H., HEILBRUN, L., KISH, J., TAPAZOUGLOU, E., JACOBS, J. & AL-SARRAF, M. (1989). Cellular DNA content parameters in untreated and recurrent squamous cell cancers of the head and neck. *Cytometry*, **10**, 334-338.

FORSTER, G., COOKE, T.G., COOKE, L.D., STANTON, P.D., BOWIE, G. & STELL, P.M. (1992). Tumour growth rates in squamous carcinoma of the head and neck measured by *in vivo* bromodeoxyuridine incorporation and flow cytometry. *Br. J. Cancer*, **65**, 698-702.

FRANZEN, G., KLINTENBERG, C., OLOFSSON, J. & RISBERG, B. (1986). DNA measurement - an objective predictor of response to irradiation? A review of 24 squamous cell carcinomas of the oral cavity. *Br. J. Cancer*, **53**, 643-651.

GREENBERG, B., WOO, L., AGUIRRE, M., BLATCHFORD, S. & GAREWELL, H. (1988). Variability of the thymidine labelling index in squamous cell carcinoma of the head and neck. *Laryngoscope*, **98**, 668-670.

HEMMER, J. (1990). *In vitro* bromodeoxyuridine labelling of squamous cell carcinomas of the oral cavity. *Eur. J. Cancer*, **26**, 113-115.

HIRANO, T., ZITSCH, R.P. & GLUCKMAN, J.L. (1991). The use of bromodeoxyuridine cytokinetic studies as a prognostic indicator of cancer of the head and neck. *Laryngoscope*, **101**, 130-133.

JOHNSON, T.S., WILLIAMSON, K.D., CRAMER, M.M. & PETERS, L.J. (1985). Flow cytometric analysis of head and neck carcinoma DNA index and S-fraction from paraffin embedded sections: comparison with malignancy grading. *Cytometry*, **6**, 461-470.

KINSELLA, T.J., RUSSO, A., MITCHELL, J.B., ROWLAND, J., JENKINS, J., SCHWADE, J., MEYERS, C.E., COLINS, J.M., SPEYER, J., KORNBLITH, P., SMITH, B., KUFTA, C. & GLADSTEIN, E.A. (1984). A phase I study of intermittent bromodeoxyuridine with conventional fractionated irradiation. *Int. J. Radiat. Biol. Phys.*, **10**, 69-76.

MITCHELL, J.B., KINSELLA, T.J., RUSSO, A., MCPHERSON, S., ROWLAND, J., KORNBLITH, P. & GLADSTEIN, E. (1983). Radiosensitization of a hematoproietic precursor cells (CFUc) in glioblastoma patients receiving intermittent intravenous infusions of bromodeoxyuridine (BUdR). *Int. J. Radiat. Oncol. Biol. Phys.*, **9**, 457-464.

PETO, R., PIKE, M.C., ARMITAGE, P., BRESLOW, N.E., COX, D.R., HOWARD, S.V., MANTEL, N., MCPHERSON, K., PETO, J. & SMITH, P.G. (1976). Design and analysis of randomised clinical trials requiring prolonged observation of each patient. *Br. J. Cancer*, **34**, 585-612.

REW, D.A., WILSON, G.D., TAYLOR, I. & WEAVER, P.C. (1991). Proliferation characteristics of human colorectal carcinomas measured *in vivo*. **78**, 60-66.

RICCARDI, A., DANOVA, M., WILSON, G., UCCI, G., DORMER, P., MAZZINI, G., BRUGNATELLI, S., GIRANO, M., MCNALLY, N.J. & ASCARI, E. (1988). Cell kinetics in human malignancies studied with *in vivo* administration of bromodeoxyuridine and flow cytometry. *Cancer Res.*, **48**, 6238-6245.

SILVESTRINI, R., MOLINARI, R., COSTA, A., VOLTERRANI, F. & GARDANI, G. (1984). Short-term variation in labelling index as a predictor of radiotherapy response in human oral cavity carcinoma. *Int. J. Radiat. Oncol. Biol. Phys.*, **10**, 965-970.

STEEL, G.G. (1977). *Growth Kinetics of Tumours*. Clarendon Press: Oxford.

TUBIANA, M. & COURDI, A. (1989). Cell proliferation kinetics in human solid tumours: relation to probability of metastatic dissemination and long term survival. *Radiother. Oncol.*, **15**, 1-18.

UICC (1984). *TNM Classification of Malignant Tumours*, 3rd edn, pp. 18-19. International Union Against Cancer: Geneva.

WILSON, G.D. (1991). Assessment of human tumour proliferation using bromodeoxyuridine - current status. *Acta Oncol.*, **30**, 903-910.